# Diagnostic accuracy of electronic surveillance tool for catheter-associated urinary tract infections in tertiary care hospitals: A meta-analysis

**DOI:** 10.1097/MD.0000000000027363

**Published:** 2021-10-01

**Authors:** Yuehua Shen, Hongmei Cui

**Affiliations:** Department of Hospital Infection Management, Huzhou Traditional Chinese Medicine Hospital Affiliated to Zhejiang Chinese Medical University, Huzhou, Zhejiang Province, PR China.

**Keywords:** catheter-associated urinary tract infections, infections, meta-analysis, tertiary care hospitals

## Abstract

**Background:**

Automated systems have been developed to reduce labor-intensive manual recordings during nosocomial infection surveillance. The diagnostic accuracies of these systems have differed in various settings.

**Methods:**

We designed this meta-analysis to evaluate the diagnostic accuracy of an electronic surveillance tool for catheter-associated urinary tract infections (CAUTIs) in tertiary care hospitals. We systematically searched databases such as Medline, Scopus, Cochrane library and Embase (from inception until November 2019) for relevant studies. We assessed the quality of trials using the diagnostic accuracy studies-2 tool, and performed a meta-analysis to obtain a pooled sensitivity and specificity for electronic surveillance. We included 6 studies with 16,492 patients in the analysis.

**Results:**

We found a pooled sensitivity of electronic diagnostic surveillance for CAUTIs of 97.5% (95% confidence interval [CI], 67.6–99.9%) and a pooled specificity of 92.6% (95% CI, 55.2–99.2%). The diagnostic odds ratio was 494 (95% CI, 89–2747). The positive likelihood ratio was 13.1 (95% CI, 1.63–105.8) and the negative likelihood ratio 0.02 (95% CI, 0.001–0.40). A bivariate box plot indicated the possibility of heterogeneity between the included studies.

**Conclusion:**

Our review suggests that electronic surveillance is useful for diagnosing CAUTIs among hospitalized patients in tertiary care hospitals due to its high sensitivity and specificity.

## 1 Introduction

The Centre for Disease Control and Prevention classified healthcare-associated infections (HAI) into 4 major types: pneumonia, surgical site infections, urinary tract infections (UTIs), and blood-stream infections.^[1]^ However, UTIs are considered the most common form of nosocomial infection accounting for about 40% of the cases.^[2]^ More than 80% of the UTIs are associated with urinary catheterization.^[3]^ Almost one-fourth of all hospitalized patients undergo urinary catheterization; and the majority (90%) of catheterized patients belong to a high-risk trauma class.^[4]^ Hence, following best practices for catheterization is essential to prevent catheter-associated urinary tract infections (CAUTIs) and reduce the caseload of nosocomial infections among these patients in tertiary care hospitals. However, many healthcare providers forget to check whether their patients have a urinary catheter in place.

Routing monitoring of variables differs based on the HAI type. According to the Centre for Disease Control and Prevention guidelines for healthcare-associated urinary tract infections, healthcare professionals should comprehensively monitor the variables related to patients’ signs, symptoms, urinary routine and culture tests, blood tests, antibiotic use, presence of invasive devices, and clinical or radiological evidence of infection during hospitalization.^[1]^ Hence, surveillance should involve bedside investigation procedures and review of medical records including care charts prepared by nurses, laboratory reports, treatment charts, radiographic examination findings, and healthcare records^[5]^; but collecting and analyzing all this information is time-consuming and costly.

Few hospitals have an established surveillance system for monitoring CAUTIs.^[3]^ Medical and health charts recorded manually by nurse practitioners communicate vital information about the patients to other members of the healthcare team. Automated nosocomial infection surveillance systems (including some for ventilator-associated pneumonia, central line-associated bloodstream infections, and device associated denominators) have been developed to reduce the labor-intensive manual recording.^[6–10]^

The diagnostic accuracies of electronic surveillance systems have been investigated in various settings with different results. However, no systematic efforts have evaluated the diagnostic accuracy of these systems. We designed this meta-analysis to evaluate the diagnostic accuracy of an electronic surveillance tool for CAUTIs in tertiary care hospitals.

## 2 Material and methods

### 2.1 Inclusion criteria

#### 2.1.1 Type of studies

We included all full-text articles examining the diagnostic accuracy of electronic surveillance tools for CAUTIs in tertiary care hospitals irrespective of their study design. The studies reported sensitivity and specificity or provided data to calculate their values. We excluded unpublished data, case reports, and studies with sample sizes smaller than 10. The ethical approval was not necessary for meta-analysis.

#### 2.1.2 Participants

The chosen studies involved patients with urinary catheterization hospitalized in a tertiary care hospital.

#### 2.1.3 Index test

The studies evaluated the electronic health record system for CAUTI surveillance.

#### 2.1.4 Reference standards

The studies used the standard manual recording of CAUTI by healthcare professionals like nurse practitioners, infection preventives, and physicians as reference standards.

#### 2.1.5 Outcome measures

Diagnostic accuracy measures included sensitivity, specificity, positive likelihood ratio (PLR), negative likelihood ratio (NLR), diagnostic odds ratio (DOR).

### 2.2 Search strategy

We performed an extensive and systematic electronic search in databases such as Medline, Scopus, Cochrane library, and Embase using medical subject headings and free-text terms like “Validation Studies,” “Urinary Tract Infections,” “Urinary Catheterization,” “Catheter-Associated Urinary Tract Infections,” “Nosocomial Infections,” “Electronic Surveillance,” “Nursing Records,” “Sensitivity,” “Specificity,” “Diagnosis,” “Manual Records,” and “Diagnostic Accuracy Studies.” The articles in the search were those from inception until November 2019 without language restrictions. We hand-searched a reference list of primary trials to find relevant articles to include in our review.

### 2.3 Selection of studies

Two authors independently performed the primary screening of titles, keywords and abstracts, and retrieved relevant full-text articles. The same authors then independently performed a secondary screening of the retrieved articles and identified the studies satisfying the inclusion criteria. Disagreements during the selection of studies were resolved through consensus.

### 2.4 Data extraction and management

The primary investigator extracted data to obtain the relevant variables from the studies. The extracted data included: study setting and design, inclusion and exclusion criteria, reference standards, index test, total participants, comorbidities, mean age, sensitivity, and specificity. We manually entered data into the STATA software (StataCorp., CollegeStation, TX). Both authors double-checked data to ensure correct entries by comparing data in the review and in the study reports.

### 2.5 Risk of bias assessment in included studies

Two authors independently evaluated the risk of bias using the quality assessment of diagnostic accuracy studies-2 tool.^[11]^ The quality assessment of diagnostic accuracy studies-2 tool consists of the following domains assessed for risk of bias and applicability: patient selection, index test, reference standard, and time interval, that is, (flow and timing) of the outcome assessments. We graded the studies as low, high, or unclear based on the presence of any bias among them.

### 2.6 Statistical analysis

We performed the meta-analysis using the STATA 14.2 software (StataCorp.). We applied the bivariate meta-analysis method to obtain the pooled estimate of diagnostic accuracy measures (sensitivity, specificity, PLR, NLR, and DOR) for the electronic surveillance system. We constructed a summary receiver operator characteristic curve (sROC) and produced a 95% predictive ellipse within the ROC space. We also calculated the area under the curve (AUC) in the sROC. The diagnostic value was better the closer the AUC value was to 1.

We generated a forest plot to graphically represent the study-specific and pooled estimates of sensitivity and specificity. We assessed heterogeneity graphically using a bivariate boxplot. We could not explore potential sources of heterogeneity with sub-group analysis or meta-regression as there were <10 studies included in the review. We used the Metandi command package for analyses.

## 3 Results

### 3.1 Selection of studies

**Figure 1:**
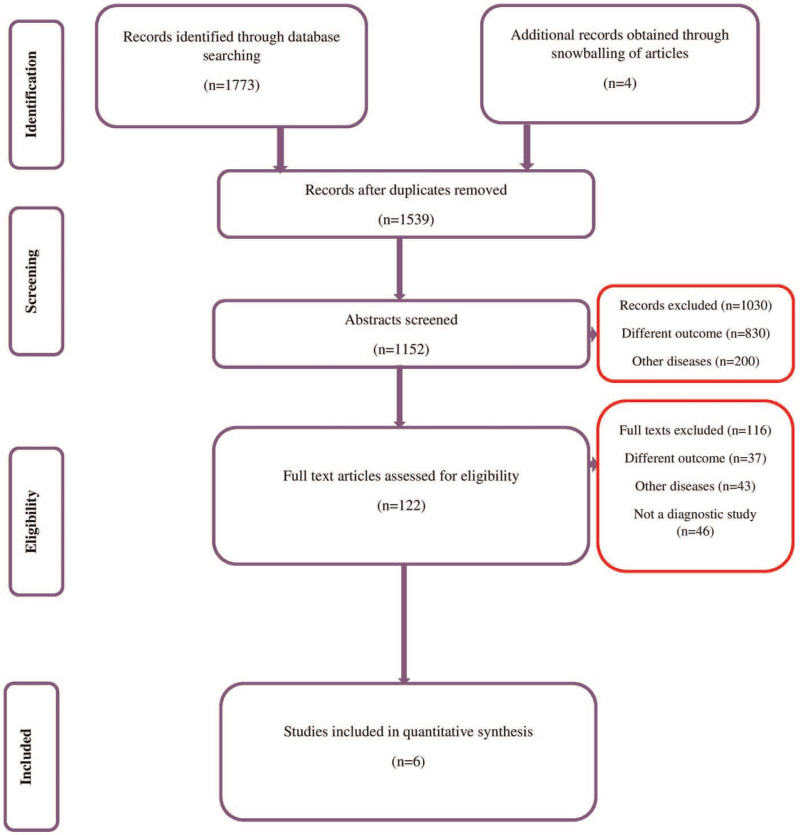
Search strategy.

Or systematic search for studies reporting the diagnostic accuracy of electronic surveillance systems for CAUTIs yielded 1773 records (673 from Medline, 547 from Scopus, 412 from Embase, and 141 from the Cochrane library). After the first screening stage (title, abstract and keywords), we retrieved 122 relevant studies. We assessed the full texts of these studies for eligibility criteria, and identified 4 potential studies from the reference list of the primary articles. Finally, we included 6 studies satisfying the inclusion criteria with 16,492 participants (Fig. 1).^[12–17]^

### 3.2 Characteristics of the studies included

Table 1 summarizes the characteristics of the included studies. Half of the studies were prospective and half retrospective. Five were conducted in the United States of America and one in Taiwan (Lo et al^[16]^). The age of the participants ranged from 55 to 72.5 years. The sample sizes of the studies varied from 175 to 11,251. All the studies had index tests as the electronic surveillance system and reference standards as manual chart/record reviews by infection preventionists, nurses, or physicians.

**Table 1:** Characteristics of the included studies (n = 6).

Study number	First author and year	Country	Study design	Sample size	Type of diagnostic modality	Gold standard comparator	Mean age (in years)
1	Branch-Elliman 2015	United States of America	Prospective cross-sectional study	2821	Electronic surveillance with natural language processing	Manual record review	Not reported
2	Choudhuri 2011	United States of America	Retrospective cohort study	204	Electronic catheter-associated urinary tract infection surveillance tool	Manual chart review	55
3	Hsu 2016	United States of America	Prospective and retrospective surveillance	175	Augmented electronic surveillance	Manual chart review by study investigators	72.5
4	Sanger 2017	United States of America	Prospective	61	Electronic surveillance with natural language processing	Manual record review	56.1
5	Sheng Lo 2013	China	Prospective	11251	Electronic surveillance	Manual chart review	Not reported
6	Wald 2014	United States of America	Retrospective	1695	Electronic health record surveillance	Manual surveillance	57.8

### 3.3 Methodological quality of the included studies

**Figure 2:**
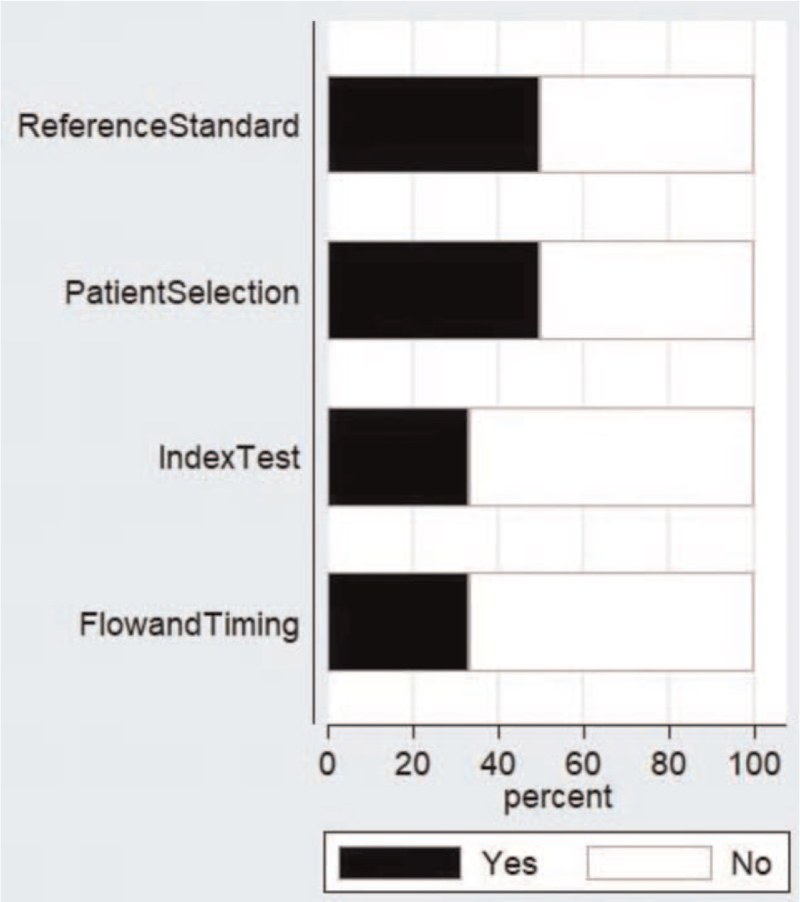
QUADAS-2 tool quality assessment results for the studies included (n = 6). QUADAS-2 = quality assessment of diagnostic accuracy studies-2.

Figure 2 depicts the risk of bias assessment for the studies in the meta-analysis. The risk of bias was high for 2 of the studies in selection bias domains. Four studies (4 out of 6) had low risk of bias in terms of the conduct and interpretation of the index test. Three had low risk of bias in terms of the conduct and interpretation of the reference standards. Finally, 2 studies had high risk of bias in relation to the patient flow and interval between index test and reference standard.

### 3.4 Diagnostic performance of electronic surveillance

**Figure 3:**
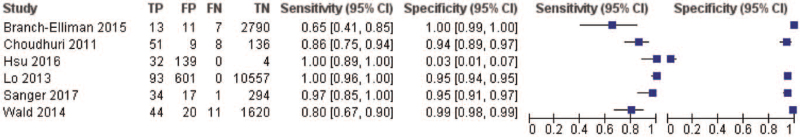
Forest plot showing pooled sensitivity and specificity for electronic surveillance during CAUTIs diagnosis. CAUTIs = catheter-associated urinary tract infections.

**Figure 4:**
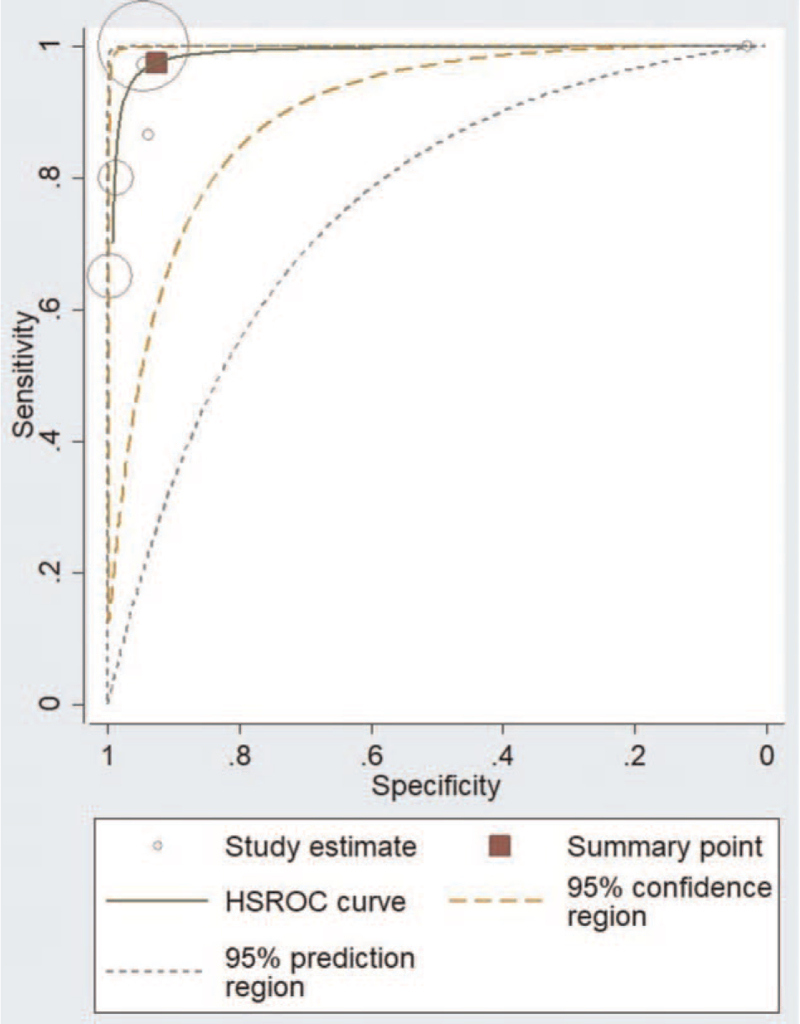
sROC curve for electronic surveillance during CAUTIs diagnosis. CAUTIs = catheter-associated urinary tract infections, sROC = summary receiver operator characteristic curve.

**Figure 5:**
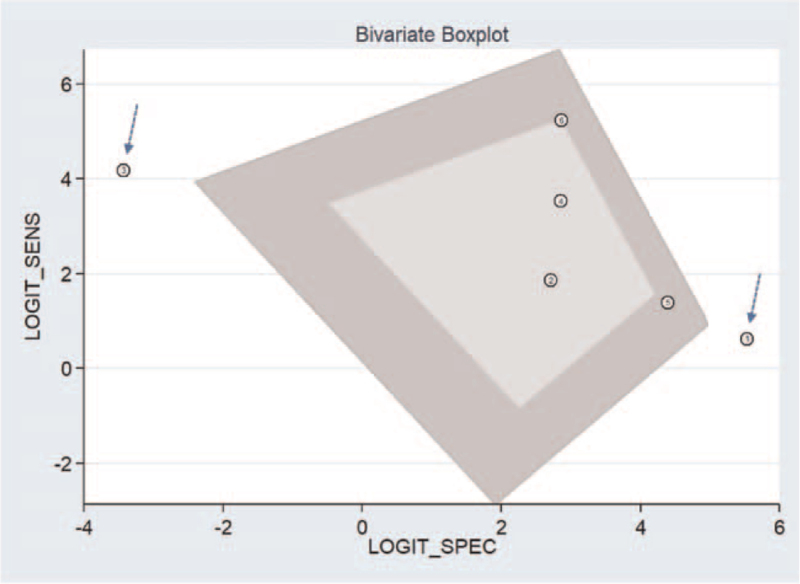
Bivariate boxplot checking the heterogeneity between the studies included.

We found 6 studies evaluating the diagnostic performance of electronic surveillance systems for CAUTIs. Figure 3 shows the forest plot with individual study sensitivities and specificities. The pooled sensitivity and specificity for electronic surveillance of CAUTI diagnoses were 97.5% (95% confidence interval [CI], 67.6–99.9%) and 92.6% (95% CI, 55.2–99.2%), respectively. The DOR was 494 (95% CI, 89–2747). The PLR was 13.1 (95% CI, 1.63–105.8) and the NLR 0.02 (95% CI, 0.001–0.40). Figure 4 depicts the sROC curve for electronic surveillance. Figure 5 shows the bivariate boxplot testing the heterogeneity between the included studies; 2 out of 6 studies are outside of the circle in the plot indicating the presence of heterogeneity between the studies. Since there were <10 studies, we could not investigate the source of heterogeneity by sub-group analysis or meta-regression. We also omitted testing for publication bias due to the low number of studies.

## 4 Discussion

CAUTIs are considered preventable, in spite of being the most common nosocomial infection in tertiary care hospitals.^[18]^ Most hospitals do not have a surveillance system to monitor the placement of urinary catheters, the duration of placement, and development of CAUTIs in hospitalized patients.^[19]^ Manual chart reviews by nurses or healthcare professionals are a labor-intensive and time-consuming process.^[20]^ Electronic surveillance is considered an effective strategy to prevent different types of HAI including CAUTIs.^[21]^ Thus, we conducted this meta-analysis to evaluate CAUTI surveillance systems and find the pooled estimates for their diagnostic performance among hospitalized patients in tertiary care hospitals.

We found a pooled sensitivity and specificity of electronic diagnostic surveillance for CAUTIs of 97.5% and 92.6%, respectively. Since this was the first review and meta-analysis on electronic surveillance for CAUTIs, we could not compare our results to others. However, we compared our findings with those of surveys of electronic surveillance systems for other forms of nosocomial infections (like those for ventilator-associated pneumonia,^[22]^ surgical site infections,^[23,24]^ and bloodstream infections^[25]^). We found that the sensitivity and specificity were highest for surgical site infection surveillance systems followed by those for CAUTIs (our data), and then by surveillance systems for bloodstream infections. Ventilator-associated pneumonia surveillance systems had showed the lowest published sensitivity (even going below the 50% range). Based on these findings, we believe the electronic surveillance systems can be applied for almost all the major HAI in tertiary care hospitals.

Our calculated PLR was 13.1 (95% CI, 1.63–105.8) and the NLR was 0.02 (95% CI, 0.001–0.40), suggesting that the CAUTI electronic surveillance system can be used as a confirmatory and exclusion tool. Our bivariate box plot indicated the possibility of heterogeneity between the studies we analyzed. However, we were not able to explore the source of heterogeneity due to the limited number of eligible studies. We also did not perform a publication bias assessment for the same reason.

The strengths of our study include its comprehensive nature, including all 6 eligible studies with 16,492 participants. This is the first review providing a pooled sensitivity and specificity for diagnostic electronic surveillance for CAUTIs. But, we are also aware of the study's limitations. First, some of the studies in our review had a high risk of bias and this may have affected our pooled estimates. Also, we found significant heterogeneity between the studies included in the meta-analysis. This limits the power of the interpretation of the pooled results. The publication bias could not be assessed due to the small number of studies included. Finally, most studies were conducted in the United States of America; and, therefore, the generalizability of our findings is limited.

However, we believe that our study provides valuable insights regarding the diagnostic accuracy of electronic surveillance systems for CAUTIs. We found that electronic surveillance systems provide both high sensitivity and specificity (>90%), and allow for confirmation and exclusion of CAUTIs with utmost certainty. Our findings suggest that automated or electronic surveillance tools with case definitions in line with international guidelines for diagnosis of CAUTIs should be implemented in tertiary care hospitals to monitor the patients with a urinary-catheter to identification and management of the infection. However, further high-quality studies in other geographical regions are needed to ensure applicability throughout the world.

## 5 Conclusion

In all, we found that diagnostic electronic surveillance is highly useful for CAUTIs among hospitalized patients due to its high sensitivity and specificity. Our results suggest that this surveillance modality can be used for CAUTI screenings in tertiary care hospitals as it is efficient and time-saving.

## Author contributions

Yuehua Shen designed the project; Yuehua Shen and Hongmei Cui were involved in data collection and data analysis; Yuehua Shen prepared the manuscript; Hongmei Cui edits the manuscript; all authors read and approved the final manuscript.

**Conceptualization:** Hongmei Cui.

**Data curation:** Yuehua Shen, Hongmei Cui.

**Formal analysis:** Yuehua Shen, Hongmei Cui.

**Investigation:** Yuehua Shen.

**Methodology:** Yuehua Shen, Hongmei Cui.

**Project administration:** Hongmei Cui.

**Supervision:** Hongmei Cui.

**Writing – original draft:** Yuehua Shen, Hongmei Cui.

**Writing – review & editing:** Yuehua Shen, Hongmei Cui.

## References

[1] Horan TC Andrus M Dudeck MA. CDC/NHSN surveillance definition of health care–associated infection and criteria for specific types of infections in the acute care setting. Am J Infect Control 2008;36:309–32. 18538699 10.1016/j.ajic.2008.03.002

[2] Klevens RM Edwards JR Richards CL. Estimating health care-associated infections and deaths in U.S. Hospitals, 2002. Public Health Rep 2007;122:160–6. 17357358 10.1177/003335490712200205 PMC1820440

[3] Saint S Kowalski CP Kaufman SR. Preventing hospital-acquired urinary tract infection in the United States: a national study. Clin Infect Dis 2008;46:243–50. 18171256 10.1086/524662

[4] Edwards JR Peterson KD Mu Y. National Healthcare Safety Network (NHSN) report: data summary for 2006 through 2008, issued December 2009. Am J Infect Control 2009;37:783–805. 20004811 10.1016/j.ajic.2009.10.001

[5] Glenister HM Taylor LJ Bartlett CLR Cooke EM Sedgwick JA Mackintosh CA. An evaluation of surveillance methods for detecting infections in hospital inpatients. J Hosp Infect 1993;23:229–42. 8099097 10.1016/0195-6701(93)90028-x

[6] Klompas M Yokoe DS Weinstein RA. Automated surveillance of health care–associated infections. Clin Infect Dis 2009;48:1268–75. 19335166 10.1086/597591

[7] Woeltje KF Butler AM Goris AJ. Automated surveillance for central line-associated bloodstream infection in intensive care units. Infect Control Hosp Epidemiol 2008;29:842–6. 18713052 10.1086/590261 PMC6788749

[8] Hota B Lin M Doherty JA. Formulation of a model for automating infection surveillance: algorithmic detection of central-line associated bloodstream infection. J Am Med Inform Assoc 2010;17:42–8. 20064800 10.1197/jamia.M3196 PMC2995624

[9] Klompas M Kleinman K Platt R. Development of an algorithm for surveillance of ventilator-associated pneumonia with electronic data and comparison of algorithm results with clinician diagnoses. Infect Control Hosp Epidemiol 2008;29:31–7. 18171184 10.1086/524332

[10] Wright M-O Fisher A John M Reynolds K Peterson LR Robicsek A. The electronic medical record as a tool for infection surveillance: successful automation of device-days. Am J Infect Control 2009;37:364–70. 19269712 10.1016/j.ajic.2008.11.003

[11] Whiting PF Rutjes AW Westwood ME. QUADAS-2: a revised tool for the quality assessment of diagnostic accuracy studies. Ann Intern Med 2011;155:529–36. 22007046 10.7326/0003-4819-155-8-201110180-00009

[12] Branch-Elliman W Strymish J Kudesia V Rosen AK Gupta K. Natural language processing for real-time catheter-associated urinary tract infection surveillance: results of a pilot implementation trial. Infect Control Hosp Epidemiol 2015;36:1004–10. 26022228 10.1017/ice.2015.122

[13] Choudhuri JA Pergamit RF Chan JD. An electronic catheter-associated urinary tract infection surveillance tool. Infect Control Hosp Epidemiol 2011;32:757–62. 21768758 10.1086/661103

[14] Hsu HE Shenoy ES Kelbaugh D. An electronic surveillance tool for catheter-associated urinary tract infection in intensive care units. Am J Infect Control 2015;43:592–9. 25840717 10.1016/j.ajic.2015.02.019 PMC4457697

[15] Sanger PC Granich M Olsen-Scribner R. Electronic surveillance for catheter-associated urinary tract infection using natural language processing. AMIA Annu Symp Proc 2018;2017:1507–16. 29854220 PMC5977673

[16] Lo YS Lee WS Liu CT. Utilization of electronic medical records to build a detection model for surveillance of healthcare-associated urinary tract infections. J Med Syst 2013;37:9923. 23321977 10.1007/s10916-012-9923-2

[17] Wald HL Bandle B Richard A Min S. Accuracy of electronic surveillance of catheter-associated urinary tract infection at an academic medical center. Infect Control Hosp Epidemiol 2014;35:685–91. 24799645 10.1086/676429

[18] Jain M Dogra V Mishra B Thakur A Loomba PS. Knowledge and attitude of doctors and nurses regarding indication for catheterization and prevention of catheter-associated urinary tract infection in a tertiary care hospital. Indian J Crit Care Med 2015;19:76–81. 25722548 10.4103/0972-5229.151014 PMC4339908

[19] Meddings J Rogers MAM Krein SL Fakih MG Olmsted RN Saint S. Reducing unnecessary urinary catheter use and other strategies to prevent catheter-associated urinary tract infection: an integrative review. BMJ Qual Saf 2014;23:277–89. 10.1136/bmjqs-2012-001774 PMC3960353 24077850

[20] Hu Z Melton GB Moeller ND. Accelerating chart review using automated methods on electronic health record data for postoperative complications. AMIA Annu Symp Proc 2017;2016:1822–31. 28269941 PMC5333220

[21] Ranji SR Shetty K Posley KA. Introduction. 2007;US: Agency for Healthcare Research and Quality, Available at: https://www.ncbi.nlm.nih.gov/books/NBK43986/. Accessed January 1, 2020. 20734530

[22] Fan Y Gao F Wu Y Zhang J Zhu M Xiong L. Does ventilator-associated event surveillance detect ventilator-associated pneumonia in intensive care units? A systematic review and meta-analysis. Crit Care 2016;20:338. 27772529 10.1186/s13054-016-1506-z PMC5075751

[23] Chalfine A Cauet D Lin WC. Highly sensitive and efficient computer-assisted system for routine surveillance for surgical site infection. Infect Control Hosp Epidemiol 2006;27:794–801. 16874638 10.1086/506393

[24] Inacio MCS Paxton EW Chen Y. Leveraging electronic medical records for surveillance of surgical site infection in a total joint replacement population. Infect Control Hosp Epidemiol 2011;32:351–9. 21460486 10.1086/658942

[25] Tseng Y-J Wu J-H Lin H-C. A web-based, hospital-wide health care-associated bloodstream infection surveillance and classification system: development and evaluation. JMIR Med Inform 2015;3:e31. 26392229 10.2196/medinform.4171 PMC4705006

